# Brain metastasis from large cell neuroendocrine carcinoma of the prostate: A case report and literature review

**DOI:** 10.1016/j.ijscr.2020.02.017

**Published:** 2020-02-11

**Authors:** Sultan Ibrahim Aljarba, Maradi Murad, Mohammed Bafaquh, Wafaa Alshakweer

**Affiliations:** a King Abdullah International Medical Research Center, Riyadh, Saudi Arabia; b College of Medicine, King Saud bin Abdulaziz University for Health Sciences, Riyadh, Saudi Arabia; c King Abdulaziz University, Jeddah, Saudi Arabia; d Pathology and Clinical Laboratory Medicine Administration, King Fahad Medical City, Riyadh, Saudi Arabia; e Department of Neurosurgery, King Fahad Medical City, Riyadh, Saudi Arabia

**Keywords:** Urology, Neurosurgery, Prostate cancer, Neuroendocrine carcinoma, CNS cancer, Brain metastasis

## Abstract

**Introduction:**

Large-cell neuroendocrine carcinoma (LCNEC) of the prostate is a rare type of prostate cancer. Only eighteen case reports have been published in the literature to date. The present case report is the first case in the literature to describe brain metastasis of LCNEC of the prostate with neuroimaging, gross, and microscopic evaluation with immunohistochemistry

**Case presentation:**

A 79-year old male with a history of high-grade prostatic adenocarcinoma treated with androgen deprivation therapy (ADT) who presented after remission with a severe headache and limbs weakness. Neuroimaging showed large right frontal lesion that caused a mass effect. Tumor resection was done, and the biopsy showed LCNEC of prostatic origin. The patient survived for 40 days after the diagnosis and tumor removal.

**Discussion:**

We discuss the spectrum of neuroendocrine differentiation in prostate carcinomas and the possible pathological pathways leading to the development of LCNEC of the prostate, and how it affects the presentation and the pattern of metastasis.

**Conclusion:**

This case report describes a brain metastasis of a rare aggressive type of prostate cancer with poor prognosis. With metastatic lesions of prostatic adenocarcinoma, the probability of NE transdifferentiation increases. LCNEC has a poor prognosis attributed to its nature and late diagnosis. Thus, reporting and investigating such tumor will positively contribute to better management for future patients

## Highlights

• Large-cell neuroendocrine carcinoma (LCNEC) of the prostate is a rare type of prostate cancer.
• Long-term ADT for conventional adenocarcinomas can induce transdifferentiation to LCNEC of the prostate.
• LCNEC can arise de novo by direct malignant transformation of NE cells of the prostate with no prior history of ADT.
• The pattern of metastasis of LCNEC resembles the pattern seen in conventional prostatic adenocarcinoma.
• The late diagnosis, the age of the patient, and the co-morbidities had worsened the prognosis of LCNEC of the prostate.

## 1 Introduction

Large-cell neuroendocrine carcinoma (LCNEC) of the prostate is an exceptionally rare type of prostate cancer. Only eighteen case reports have been published in the literature to date [[1], [2], [3], [4]]. LCNEC of the prostate is very aggressive and associated with widespread metastases [4,5]. The commonly reported sites of metastasis are lymph nodes, lungs, bones, and visceral organs, especially the liver [4,5]. Brain metastasis of LCNEC of the prostate was only reported in two cases published by Evans et al. but no neuroimaging, gross, and microscopic evaluation of the brain lesion was published [4]. To date and in line with the SCARE criteria, the present case report is the first case in the literature to describe brain metastasis of LCNEC of the prostate with neuroimaging, gross, and microscopic evaluation with immunohistochemistry [6].

## 2 Case presentation

A 79 years old male presented to the neurosurgery clinic complaining of a headache and dizziness with upper and lower limb weakness for the past 8 months and urinary incontinence for the past 2 months. The patient was a known case of hypertension and diabetes with a history of prostatic adenocarcinoma with lung metastasis. The prostate biopsy showed high-grade prostatic adenocarcinoma with a Gleason score (4 + 5 = 9), and he was treated by androgen deprivation therapy (ADT) nine years ago. Two years ago, a follow-up bone scan and computed tomography (CT) scan of the chest, abdomen, and pelvis showed that the lung lesion disappeared, and no other metastasis was found. On physical examination, the patient was alert and oriented to time, place, and person with GCS 15/15, the pupils were equal and bilaterally reactive, and the power was 4/5 in both upper and lower limbs. At the time of admission, the total prostate-specific antigen (PSA) and free-PSA levels were 12.3 ng/mL and 1.8 ng/mL, respectively.

**Fig. 1:**
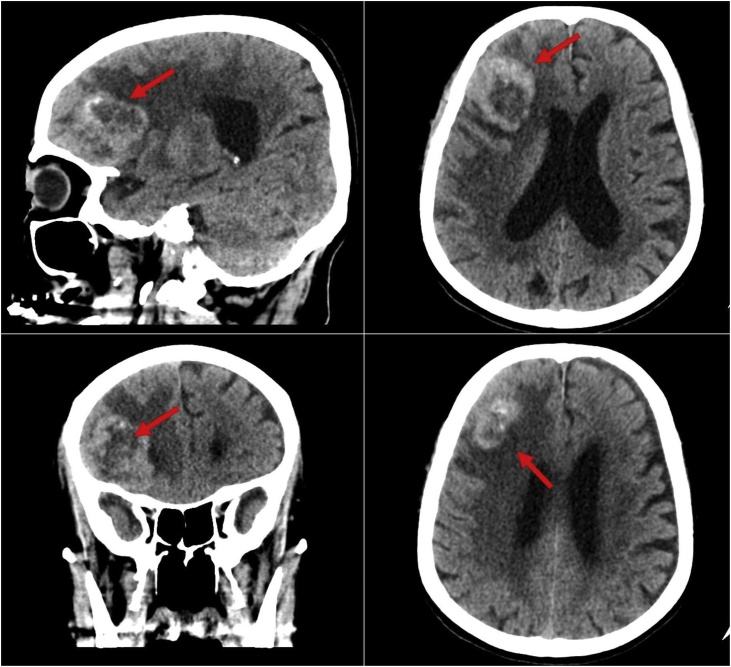
A large right frontal lobulated peripherally hyper-attenuating mass with punctuate foci of calcification and central hypodensity, probably representing necrotic component. The mass measures 4.5 × 4 × 3.5 cm in AP, CC and transverse dimensions, respectively. It was surrounded by vasogenic edema and causing a mass effect on the right frontal horn with mild leftward midline shift by 5 mm.

**Fig. 2:**
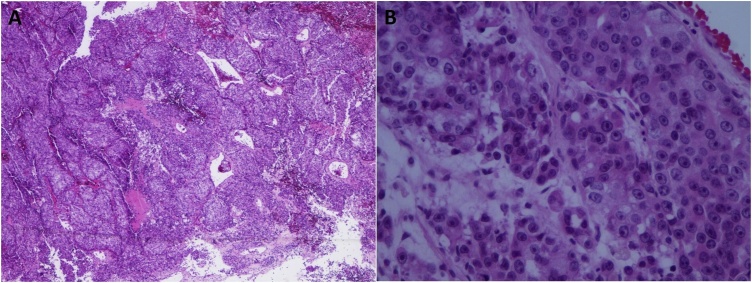
Hematoxylin and eosin stain of the tumor with a low-power view (4×) shows irregular nests of epithelium with foci of necrosis (A) and a high-power view (40×) showing large cells with large nuclei and inclusion-like nucleoli (B).

**Fig. 3:**
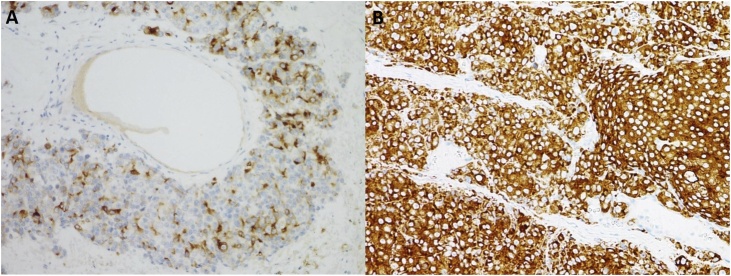
Immunohistochemical staining shows a focal cytoplasmic positivity for prostatic specific antigen (A) and synaptophysin granular cytoplasmic positivity (B).

Unenhanced CT scan of the brain revealed a large right frontal lobulated peripherally hyper-attenuating mass with punctuating foci of calcification and central hypodensity which was surrounded by vasogenic edema, and it was causing a mass effect on the right frontal horn with mild leftward midline shift (Fig. 1). Contrast-enhanced CT scan of the chest, abdomen, and pelvis did not show any metastatic lesions. The lesion was removed by a modified pterional and orbital osteotomy approach. The gross evaluation of the tumor revealed a grey-tan mass measuring 5.5 × 4.5 × 2 cm with a lobulated outer surface and an attached strap of dura mater. The serial sectioning of the mass showed areas of necrosis. The histopathological examination with hematoxylin and eosin staining showed a monomorphic infiltrate of large cells with prominent nuclei and ill-defined cytoplasmic membrane, arranged in variable size nests with evident necrosis and a mitotic rate more than 4/10 high-power fields (Fig. 2). Immunohistochemical study (IHC) of the tumor showed positive reaction with EMA, Cam 5.2, synaptophysin, PSA and AMACAR antibodies and the tumor cells were negative for S100, TTF-1, CK7, CK20 and CDX2 (Fig. 3). The histopathological examination and focal positivity for PSA, AMACR, and synaptophysin (NE marker) supported the diagnosis of LCNEC of prostatic origin.

Postoperative magnetic resonance imaging (MRI) showed no definite enhancing of a residual tumor with postoperative edema and hemorrhage. In the postoperative period, the patient had meningitis and minor surgical site infection, and cerebrospinal fluid (CSF) analysis showed low glucose levels and high protein levels with a negative CSF culture. In the fourth day after the surgery, the patient developed tachycardia, and an echocardiogram was done revealing severe aortic stenosis. Due to the patient age and co-morbidities, no intervention was commenced, and the patient was started on aspirin 81 mg daily. In the fifth day after the surgery, the patient developed a decreased level of consciousness with GCS 9/15, and a CT scan showed dilated ventricles. Due to a raised intracranial pressure (ICP), an external ventricular drain (EVD) was inserted. A follow-up CT scan showed normal ventricular size, and ICP measurement was normal. However, patient neurological status did not improve. The patient was referred to palliative care due to his multiple co-morbidities, and the poor clinical status. The patient passed away as a result of a cardiac arrest 43 days after the surgery.

## 3 Discussion

The spectrum of neuroendocrine (NE) differentiation in prostate carcinomas can be classified according to 2016 WHO classification depending on the pathophysiology and the molecular aspects of the disease [5]. NE differentiation can be found as a focal differentiation in the usual acinar or ductal adenocarcinoma of the prostate which is identifiable by immunohistochemical staining [5]. Carcinoid tumor of the prostate shows a well-differentiated NE tumor occurring in the prostate gland [5]. Small cell NE differentiation is a high-grade tumor of the prostate which is defined by distinctive nuclear features such as the lack of prominent nucleoli, nuclear molding, and crush artifacts [5]. LCNEC is a high-grade NE tumor with distinctive morphologic criteria of non–small cell carcinomas consisting of large nests with peripheral palisading, large cell size, abundant cytoplasm, prominent nucleoli, vesicular clumpy chromatin, and frequent necrosis accompanied by a high mitotic rate and positive immunohistochemical staining with at least one NE marker (synaptophysin, chromogranin, CD56) [5]. NE differentiation in prostate carcinomas is very rare, representing 1%–5% of all cases of prostate cancer [4]. LCNEC is exceptionally rare compared to other NE tumors of the prostate, and it is limited to sporadic case reports and case series [4,5]. The largest case series was presented by Evans et al. where he discussed the pathological manifestations and the pattern of metastasis [4].

LCNEC can emerge from two possible pathological pathways. First, in patients treated with long-term ADT for conventional adenocarcinomas in a process known as transdifferentiation [7]. In vitro studies of the prostate cancer cell line LNCaP revealed a reduction in androgen receptor expression in the cultures grown with the absence of androgens [7]. This mechanism is consistent with what is observable in some clinical cases including our case, where there was a history of long-term ADT which posed a selection pressure on non-NE tumor cells from the conventional adenocarcinoma resulting in evolution and clonal proliferation and emergence of NE carcinomas with hormone-refractory status. Castrate-resistance was observed in our patient, as the serum PSA raised and the brain metastasis appeared, despite treatment. Interestingly, it has been proposed that nonmalignant NE cells of the prostate under adrenogenic depurative environment can promote androgen-independent growth of non-NE tumor cells in a paracrine fashion by secreting growth-promoting neuropeptides [7]. Although the evidence for LCNEC transdifferentiation is still obscure, the existence of mixed NE carcinoma-acinar adenocarcinoma is one of the strongest evidence of transdifferentiation [4,5]. Another evidence is the presence of mixed features between the LCNEC and conventional adenocarcinoma such as co-expression of NE markers and PSA which indicate the presence of intermediate forms of tumor cells and would further support the process of transdifferentiation [4]. Interestingly, our patient’s tumor IHC showed focal positivity for PSA, AMACR, and synaptophysin that showed mixed features between the LCNEC and conventional adenocarcinoma. One of the most interesting results in animal models that also supports the process of transdifferentiation is probasin-large T antigen (Tag) transgenic mouse line that developed prostatic adenocarcinoma with progressive NE differentiation with advancing age, and metastasis that showed histological features and IHC of NE differentiation [8]. Second, LCNEC can arise de novo by direct malignant transformation of NE cells of the prostate with no prior history of ADT. This mechanism was observed in a few case reports [[1], [2], [3], [4]]. The relation between ADT and the progression of adenocarcinoma to NE carcinoma is still poorly understood, and it needs further investigation. It is clear that the development of NE differentiation, small cell or LCNEC type, occur in a minority of patients and the spectrum of factors that play a role in the development of such an aggressive tumor is yet to be discovered.

Our patient presented with elevated levels of serum PSA. Well-differentiated NE tumors (Carcinoid tumors) of the prostate do not express or secrete PSA [5]. However, it was observed that LCNEC expresses PSA variably ranging from complete negative staining to focal positivity with variable levels of serum PSA [1,4,5]. This variability could be attributed to the presence of intermediate forms of cells with mixed features between the LCNEC and conventional adenocarcinoma, the effect of treatment, and most importantly, the vague definition and poor understanding of LCNEC.

The pattern of metastasis of LCNEC resembles the pattern seen in conventional prostatic adenocarcinoma demonstrating a preference for lymph nodes, lungs, bones, and liver [4]. Only two cases of brain metastasis from LCNEC have been reported in the literature by Evans et al. case series [4]. Brain metastasis from NE tumors is extremely rare. The incidence of brain metastasis in a patient diagnosed with NE tumors is estimated to be 1.5–5% and brain metastasis from NE tumors represent 1.3–1.4% of all patients with brain metastasis [9]. However, the pattern of metastasis and likelihood of metastasis in a patient with LCNEC of the prostate should be further investigated. It is clear that the LCNEC of the prostate is a rare disease, and metastasis to the brain is also a rare event. Our patient presented with a headache and dizziness with upper and lower limb weakness with urinary incontinence and neuroimaging showed large right frontal mass. The brain mass was highly suspicious for metastasis and raised other possible diagnoses such as high-grade meningioma or glioma. Surgical removal of the tumor is the method of choice for large and symptomatic single brain metastasis providing quick symptomatic relief [10]. Post-operatively the patient had a tachycardia as a result of an unexpected severe aortic stenosis which was diagnosed after the surgery. The patient did not tolerate the procedure very well, mainly due to his age and co-morbidities. The patient developed a decreased level of consciousness and dilated ventricles, and despite adequate treatment, the patient neurological status did not improve. The patient was made a DNR and referred to palliative care. The patient survived for 43 days after the surgery.

## 4 Conclusion

Patients’ complaints such as weakness, headache, altered consciousness, or focal deficits should be promptly investigated with detailed neurological history, physical examination, and neuroimaging.

The late diagnosis, the age of the patient, and the co-morbidities had worsened the prognosis of LCNEC of the prostate. It is very clear that early detection and early treatment of metastatic LCNEC of the prostate would dramatically improve outcomes as the metastasis and progression of NE transdifferentiation are associated with the environment and age of the tumor.

## Declaration of Competing Interest

The authors declare that we have no conflict of interest.

## Sources of funding

This study did not receive any funding support.

## Ethical approval

This is a case report; therefore, it did not require ethical approval from ethics committee.

## Consent

The patient is deceased.

Written informed consent was obtained from the patient next of kin for publication of this case report.

## Author contribution

Sultan Ibrahim Aljarba: wrote and edited the manuscript.

Maradi Murad: contributed to the writing and literature review.

Mohammed Bafaquh and Wafa Shakweer were the attending doctors for the patient and they helped edit the case report.

## Registration of research studies

N/A.

## Guarantor

Sultan Ibrahim Aljarba and Wafa Shakweer.

## Provenance and peer review

Not commissioned, externally peer-reviewed.
